# Metformin and Chemoprevention: Potential for Heart-Healthy Targeting of Biologically Aggressive Breast Cancer

**DOI:** 10.3389/fpubh.2020.509714

**Published:** 2020-10-29

**Authors:** Veronica C. Jones, Eric C. Dietze, Tijana Jovanovic-Talisman, Jeannine S. McCune, Victoria L. Seewaldt

**Affiliations:** City of Hope Comprehensive Cancer Center, Duarte, CA, United States

**Keywords:** breast cancer, prevention, metformin, chemoprevention, diabetes, heart disease

## Abstract

Currently, tamoxifen is the only drug approved for reduction of breast cancer risk in premenopausal women. The significant cardiovascular side effects of tamoxifen, coupled with lack of a survival benefit, potential for genotoxicity, and failure to provide a significant risk-reduction for estrogen receptor-negative breast cancer, all contribute to the low acceptance of tamoxifen chemoprevention in premenopausal women at high-risk for breast cancer. While other prevention options exist for postmenopausal women, there is a search for well-tolerated prevention agents that can simultaneously reduce risk of breast cancers, cardiovascular disease, and type-2 diabetes. Metformin is a well-tolerated oral biguanide hypoglycemic agent that is prescribed worldwide to over 120 million individuals with type-2 diabetes. Metformin is inexpensive, safe during pregnancy, and the combination of metformin, healthy lifestyle, and exercise has been shown to be effective in preventing diabetes. There is a growing awareness that prevention drugs and interventions should make the “whole woman healthy.” To this end, current efforts have focused on finding low toxicity alternatives, particularly repurposed drugs for chemoprevention of breast cancer, including metformin. Metformin's mechanisms of actions are complex but clearly involve secondary lowering of circulating insulin. Signaling pathways activated by insulin also drive biologically aggressive breast cancer and predict poor survival in women with breast cancer. The mechanistic rationale for metformin chemoprevention is well-supported by the scientific literature. Metformin is cheap, safe during pregnancy, and has the potential to provide heart-healthy breast cancer prevention. On-going primary and secondary prevention trials will provide evidence whether metformin is effective in preventing breast cancer.

## Current Breast Cancer Prevention Strategies

Currently, tamoxifen is the only drug approved for reducing risk of breast cancer in premenopausal women. The approval of tamoxifen was based on the first National Surgical Adjuvant Breast and Bowel Project (NSABP) Breast Cancer Prevention Trial (P1) (1, 2). The P1 trial demonstrated that high-risk women who took tamoxifen had a “50% decrease in the incidence of estrogen receptor-positive breast cancer” (1). Results from the P1 trial underlined the decision of the US Food and Drug Administration (FDA) in October 1998 to approve tamoxifen as a chemoprevention agent for premenopausal high-risk women.

In 2013, the risk reduction benefit of tamoxifen was also shown in a meta-analysis of four randomized controlled trials (3): (1) Royal Marsden (4, 5), (2) International Breast Cancer Intervention Study (IBIS-1) (6, 7), (3) P1 (1, 2), and (4) Italian Randomized Tamoxifen Trial (8, 9). This analysis showed a 33% reduction (*p* < 0.0001) in all breast cancers (10, 11) in high-risk women who took tamoxifen chemoprevention vs. placebo controls (3). As in the P1 trial, the observed reduction was primarily due a decrease in the numbers ER-positive breast cancer (44% in invasive breast cancers (*p* < 0.0001) and DCIS (*p* = 0.009). Although tamoxifen-prevention was given for 5-years, follow-up evaluation of the high-risk subjects provide evidence that the long-term risk-reduction in subjects who took tamoxifen may persist up to 10 years (3).

The benefit of tamoxifen appears to be in risk-reduction of ER+ breast cancer; tamoxifen has failed to demonstrate in high-risk women (1) a significant risk reduction for ER- breast cancer and (2) a survival benefit. An extended analysis (median 16 years) of IBIS-I study participants, continues to shows in the tamoxifen vs. placebo arms “no difference in the number of breast cancer deaths (*p* = 0.8)” (12).

Despite initial recommendations by the FDA and American Society for Clinical Oncology, very few women take tamoxifen (11); it is estimated that only 5–12% of women offered tamoxifen chemoprevention elect to take tamoxifen (11).

Tamoxifen has been shown to increase risk for cardiovascular events, including venous thrombosis, pulmonary embolism, and stroke, and increases risk for endometrial cancer (12–14). Other side effects of tamoxifen include hot flashes, dyspareunia, depression, cataracts, weight gain, and bone loss in premenopausal women (12–15). Consistent with the increased risk of endometrial cancer in humans, a 2013 study in rats showed that 13-week tamoxifen treatment increased DNA point mutations in the liver (16). Lastly, a concern was raised that tamoxifen may be less active in the 5–10% of individuals who carried homozygous variant of the *CYP2D2* gene; this gene variant has low activity to convert tamoxifen to its more active metabolite, 4-hydroxytamoxifen. Lacking in the analysis was a consideration of the concentration of 4-hydroxytamoxifen required to saturate ER; consequently, prospective clinical studies did not demonstrate a reduction in tamoxifen efficacy in individuals with the *CYP2D2* variant (17).

While tamoxifen is the only agent approved for breast cancer prevention in premenopausal women, other agents have been approved for postmenopausal women. In the NSABP Study of Tamoxifen and Raloxifene (STAR) trial (raloxifene 60 mg vs. tamoxifen 20 mg), raloxifene was shown to reduce the incidence of breast cancer in postmenopausal women (18). Raloxifene does not increase the risk of endometrial cancer, however, the incidence of ischemic heart disease and stroke was equivalent to the risk associated with tamoxifen (18). IBIS-II tested anastrozole (1.0 mg) vs. placebo in postmenopausal women; the study found a significant decrease in breast cancer in women who took anastrozole; there was no increased incidence of fractures or cardiovascular disease (19). In the Mammary Prevention.3 trial (MAP.3) exemestane (25 mg) vs. placebo in postmenopausal women was associated with a decreased incidence of both ductal carcinoma *in situ* and invasive breast cancer; with a median follow-up of 3 years, side effects and impact on quality of life were minimal (20).

## Need for Heart-Healthy Breast Cancer Chemoprevention

Women are not just at risk for breast cancer but also face the risk of developing heart disease, obesity, and type-2 diabetes. Furthermore, with the risk of currently available chemoprevention agents potentiating cardiovascular disease, there is a need to identify agents that can effectively target both conditions: breast cancer and cardiovascular disease. To this end, current efforts have focused on finding alternative prevention strategies that have the potential to reduce not just breast cancer but also reduce the risk for cardiometabolic diseases. Potential strategies have included exercise, aspirin, and metformin.

### Metformin

Metformin (1,1-dimethylbiguanide hydrochloride) is a well-tolerated oral agent that is prescribed for first-line treatment of type-2 diabetes (21, 22) and is approved for treatment of polycystic ovary and gestational diabetes (23). Metformin is well-tolerated by the majority of patients; common metformin side effects include lack of appetite, epigastric pain, nausea, and diarrhea (24). The most significant potential side effect is lactic acidosis; consequently, metformin is not prescribed in individuals with kidney and/or liver disease (23, 25). The mechanism of action of metformin remains a topic of current investigations. It is accepted that metformin inhibits hepatic gluconeogenesis and decreases intestinal absorption of glucose, secondarily decreasing circulating insulin (21, 26). Metformin is also thought to indirectly increase insulin sensitivity by increasing peripheral glucose utilization (21).

Until recently, most clinical care has focused on treatment of type-2 diabetes rather than its prevention. However, several well-controlled studies have shown that it is possible to prevent type-2 diabetes through a combination of diet, exercise, and metformin. The Diabetes Prevention Program/Diabetes Prevention Program Outcomes Study (DPP/DPPOS) is the largest and longest clinical trial of metformin for the prevention of type-2 diabetes (27, 28). Study participants in the DPP/DPPOS cohort have over 15 years prospective assessment of the impact of metformin and lifestyle modification on type-2 diabetes, cardiovascular events, safety, and fiscal outcomes (27). Metformin and intensive lifestyle modification resulted in a 50% type-2 diabetes risk-reduction in women with a history of type-2 diabetes (29). Based on findings from the DPP/DPPOS study, in 2014, the American Diabetes Association (ADA) published formal recommendations for prevention of type-2 diabetes (30). Recommendations included: (1) individuals with impaired glucose tolerance or a HgbA1c 5.7–6.4 should be referred to a life-style modification (7% weight loss target) and moderate physical activity (e.g., walking) for 150 min/week (30). These recommendations may also prove beneficial in modifying breast cancer risk; as outlined below, metformin is undergoing testing for primary and secondary breast cancer prevention.

### Metformin and Breast Cancer: Epidemiology Studies

Population-based studies provide evidence that cancer incidence and mortality decreased in individuals with cancer who took metformin (31–33). In a retrospective study of women with breast cancer who received neoadjuvant chemotherapy individuals who took metformin had a higher rate of pathologic complete remission vs. those did not [24 vs. 8%, *p* = 0.007; (34)]. In a 2014 meta-analysis, individuals who took metformin had a lower incidence of breast cancer (SRR = 0.94; 95% CI, 0.90–0.99) (35). These epidemiologic studies represent a starting point for recent prospective clinical trials testing the impact of metformin on primary and secondary breast cancer prevention.

Epidemiology studies investigating the impact of metformin on breast cancer incidence are limited by several factors. These factors include: (1) racial and ethnic differences in body mass index (BMI), (2) inability of BMI to precisely identify individuals who are metabolically unhealthy, and (3) the heterogeneity of breast cancer as a disease. A BMI ≥30 kg/m^2^ is the most frequently used measure of adiposity (36). BMI is an inexact measure of risk, particularly when comparing individuals of different race and ethnicity. Muscle tissue weighs significantly more per unit volume than adipose tissue; consequently fit, muscular individuals can be mistakenly identified as overweight (BMI 25–30 kg/m^2^) or obese.

BMI is not a precise measure of metabolic health. Over the past 20 years, the observation has been made that some individuals with a BMI > 30 kg/m^2^ are metabolically healthy, “metabolically healthy obese” (37). In contrast to individuals who are obese but metabolically healthy, there are also individuals with a normal BMI (BMI <25 kg/m^2^) who have abnormal metabolic profiles and are at increased risk for cardiovascular disease and type-2 diabetes. Current definition of metabolically unhealthy individuals with a normal BMI includes (1) BMI <25 kg/m^2^, (2) insulin-resistance, hypertriglyceridemia, (3) abdominal fat distribution, and (4) elevated blood pressure (37).

## Type-2 Diabetes, Metformin, and Breast Cancer Subtypes

Type-2 diabetes is well-established to increase a woman's risk of developing breast cancer. The association between Type-2 diabetes and breast cancer subtypes, however, remains a work in progress, particularly since the majority of studies are underpowered. A case-control study of 916 postmenopausal women with breast cancer cases and 1,094 population-based controls conducted by Garcia-Esquinas et al. found that type-2 diabetes was associated with a 2.25-fold increased risk for triple negative breast cancer (TNBC) (38); this study was limited by a low number of TNBC and the study of only postmenopausal women. The Carolina Breast Cancer Study included 225 women with TNBC; no statistical association was found between type-2 diabetes and TNBC; unfortunately, this study did not test for the association between insulin-resistance and TNBC (39). A case-case study by Lara-Medina et al. of Latinas with breast cancer (469 women with TNBC) found no statistical association between type-2 diabetes and TNBC (40).

The most complete and well-designed epidemiologic study was a retrospective multi-center population-based case-case study of 4,557 women with breast cancer ages 20–69 years old performed by Chen et al.; 1,446 women had TNBC (41). The investigators identified that women with type-2 diabetes had a 38% (95% CI: 1.01–1.89) increased odds of having TNBC (vs. women without type-2 diabetes) (41).

Interestingly, Chen et al. also found that current and extended-time metformin use (13–24 months metformin) within 2 years of diagnosis, increased the odds of a woman having TNBC (*OR* = 1.54; 95% CI: 1.07–2.22 and *OR* = 1.80; 95% CI:1.13–2.85, respectively) (41). These latter results are puzzling, given the ability of insulin to activate signaling pathways that drive the aggressive biology of TNBC and the known ability of metformin to lower circulating insulin.

**Figure 1:**
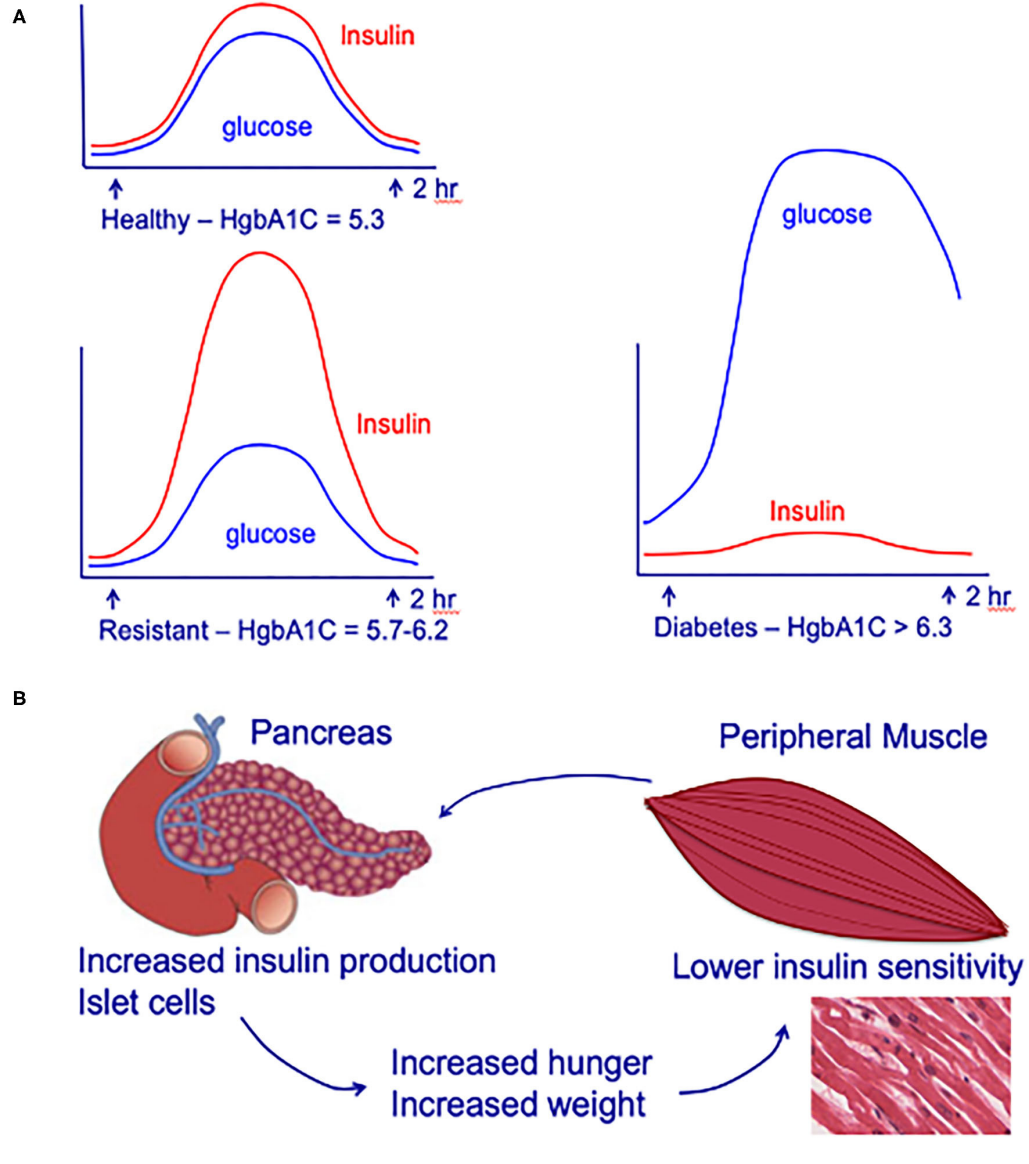
**(A)** Circulating insulin and glucose levels in healthy individuals (Healthy), insulin-resistant individuals (Resistant), and individuals with type-2 diabetes (Diabetes) at baseline and at 2 h after eating. **(B)** Impact of insulin-resistance on pancreatic islet cells, peripheral muscle, and individual. Insulin resistance in peripheral muscle tissue results in increased insulin demands from the pancreas. Increased circulating insulin drives hunger and increases weight, leading to a positive feedback loop that increases the chance of an individual developing type-2 diabetes. Adapted from (42).

Epidemiologic studies are powerful tools for generating associations but do not test mechanisms. First off, as pointed out by Chen et al., it may be that the women who had the most poorly controlled diabetes (41), were the individuals who had the longest use of metformin; HgbA1c values for these individuals were not reported. While the number of women using metformin were carefully determined, it is not clear that the investigators incorporated insulin-use (insulin-dependent type-2 diabetes) in their risk models. Furthermore, these risk models do not account for individuals with insulin-resistance (Figure 1). Ultimately, the studies by Chen et al. are extremely important because they highlight how complex the associations between metformin-use, insulin-use, and TNBC are likely to be and underscore the importance of window-of-opportunity trials and ongoing prospective metformin prevention trials (such as MA-32, described below).

## Metformin Transport and Mechanism of Action

After oral administration, the oral bioavailability is 55 ± 16% (mean ± standard deviation); metformin is predominantly absorbed in the small intestine (43). Metformin is excreted unchanged in the urine and has a half-life between 4 and 8 h (44). Metformin's absorption and renal clearance is primarily mediated by OCT2/MATE1/MATE2-K (organic cation transporter 2/multidrug and toxin extrusion 1/ multidrug and toxin extrusion 2-K) (45). There are frequent polymorphisms in OCT2, MATE1, and MATE2-K that impact clearance metformin [Table 1; (46, 62)]. Up to 9% of non-Hispanic Whites exhibit an “OCT1 null phenotype” (46). To date, there have been variable findings in pharmacogenomic studies in humans. However, there is evidence that cancer cell lines with high MATE2 expression may be resistant metformin's growth inhibitor effects (63).

**Table 1:** Select list of clinically relevant known metformin pharmacokinetic and pharmacodynamic genes.

**Gene**	**Protein**	**Effect**	**References**
*SLC22A1*	OCT1	Low-function alleles linked to less reduction in HgbA1c	(46–54)
*SLC22A2*	OCT2	Change in metformin PK; no known clinical impact	(53)
*SLC22A3*	OCT3	Changes in metformin PK; no known clinical impact	(54)
*SLC47A1*	MATE1	Alleles linked to increased reduction in HgbA1c	(47, 50, 55)
*SCLa7A2*	MATE2	Low-function alleles linked to less reduction in HgbA1c	(55, 56)
*SRR*	Serine racemase	Metabolic changes	(57)
*ATM*	ATM	Low- and high-function alleles linked to change in HgbA1c	(58–60)
*LBK/STK11*	Upstream regulator of AMPK	Decreased ovulation in women with polycystic ovarian syndrome.	(47, 61)
*PKRAA1, PKRAA2, PKRAB2*	AMPK sub-units	Incidence type-2 diabetes	(47)
*ABCC8-KNKJ11*	Subunit beta cell potassium channel	Incidence type-2 diabetes	(47)

**Figure 2:**
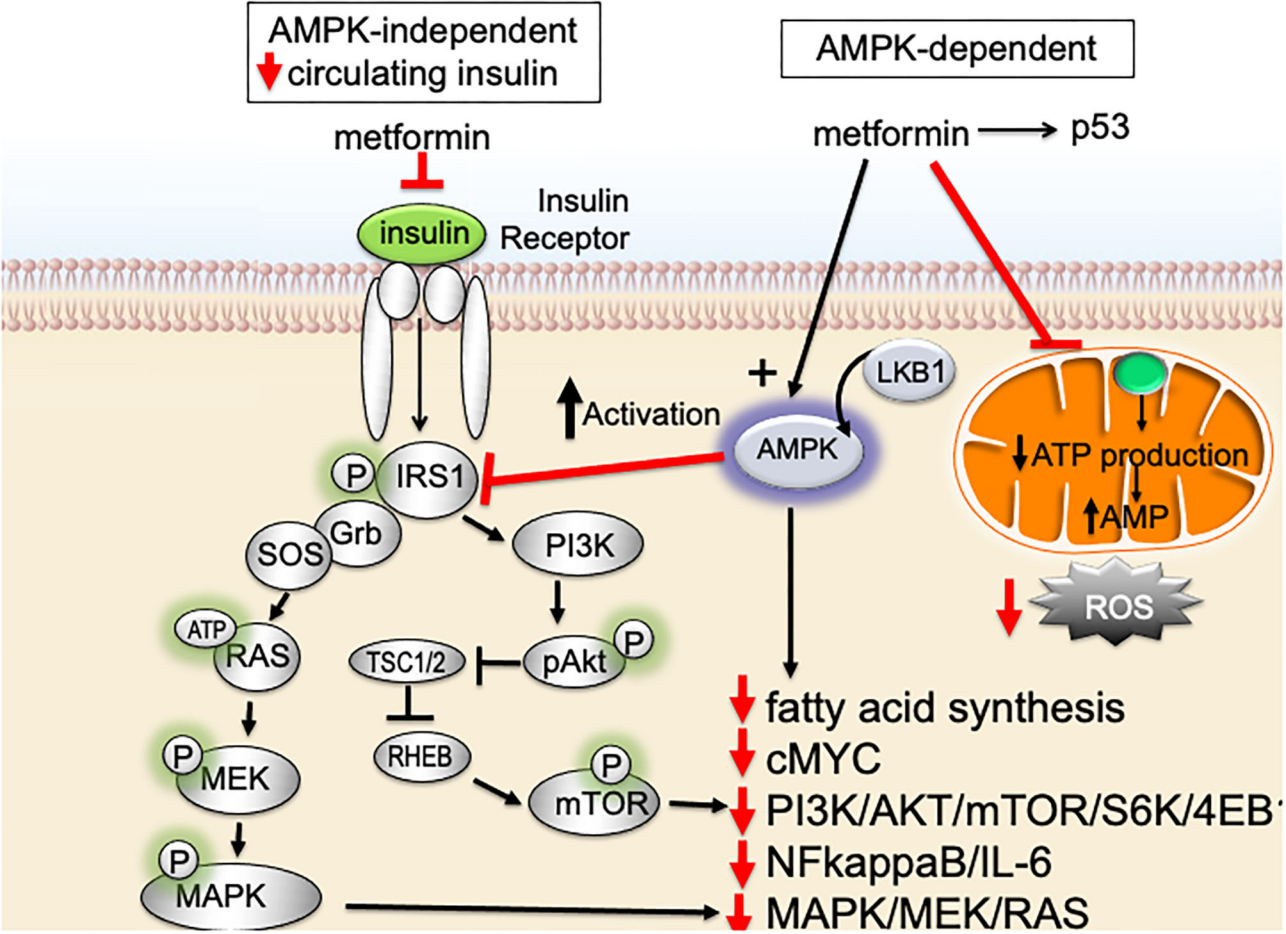
Impact of metformin on insulin-resistance, serum insulin, and signaling pathways important for breast cancer aggressive biology.

Despite metformin being one of our oldest medications, the precise molecular mechanism(s) underlying metformin's insulin-lowering effects, as well as its potential anti-neoplastic potential, are not completely understood. It is well-accepted that metformin inhibits hepatic gluconeogenesis and secondarily lowers circulating insulin. However, the precise mechanism(s) of metformin-action remains a work in progress. Two major pathways are thought to account for the main actions of metformin and metformin's proposed anti-cancer effects (Figure 2); both pathways converge on mammalian target of rapamycin (mTOR): (1) AMPK (adenosine monophosphate-activated protein kinase) independent, driven by metformin's ability to secondarily lower serum insulin and (2) AMPK-dependent, regulated by metformin-suppression inhibition of mitochondrial complex-I (complex-I).

Metformin signals via an AMPK-independent pathway; in this pathway metformin secondarily lowers circulating insulin levels and inhibits insulin/insulin-like growth factor-1 (IGF-1)-signaling. Under nutrient-rich circumstances, IGF-1 binds to the IGF-1 receptor (IGF-1R) leading to activation of (1) PI3K (phosphatidylinositol-3-kinase)/AKT/mTOR-network signaling and (2) RAS/RAF/mitogen activated protein kinase (MAPK) [Figure 2; (64)]. Activation of PI3K/MAPK-pathways increase cell proliferation and activates signaling pathways associated with aggressive cancer biology in humans. By lowering circulating insulin, metformin inhibits IGF-1/IGF-1R signaling and inhibits PI3K- and MAPK-signaling pathways (Figure 2).

**Figure 3:**
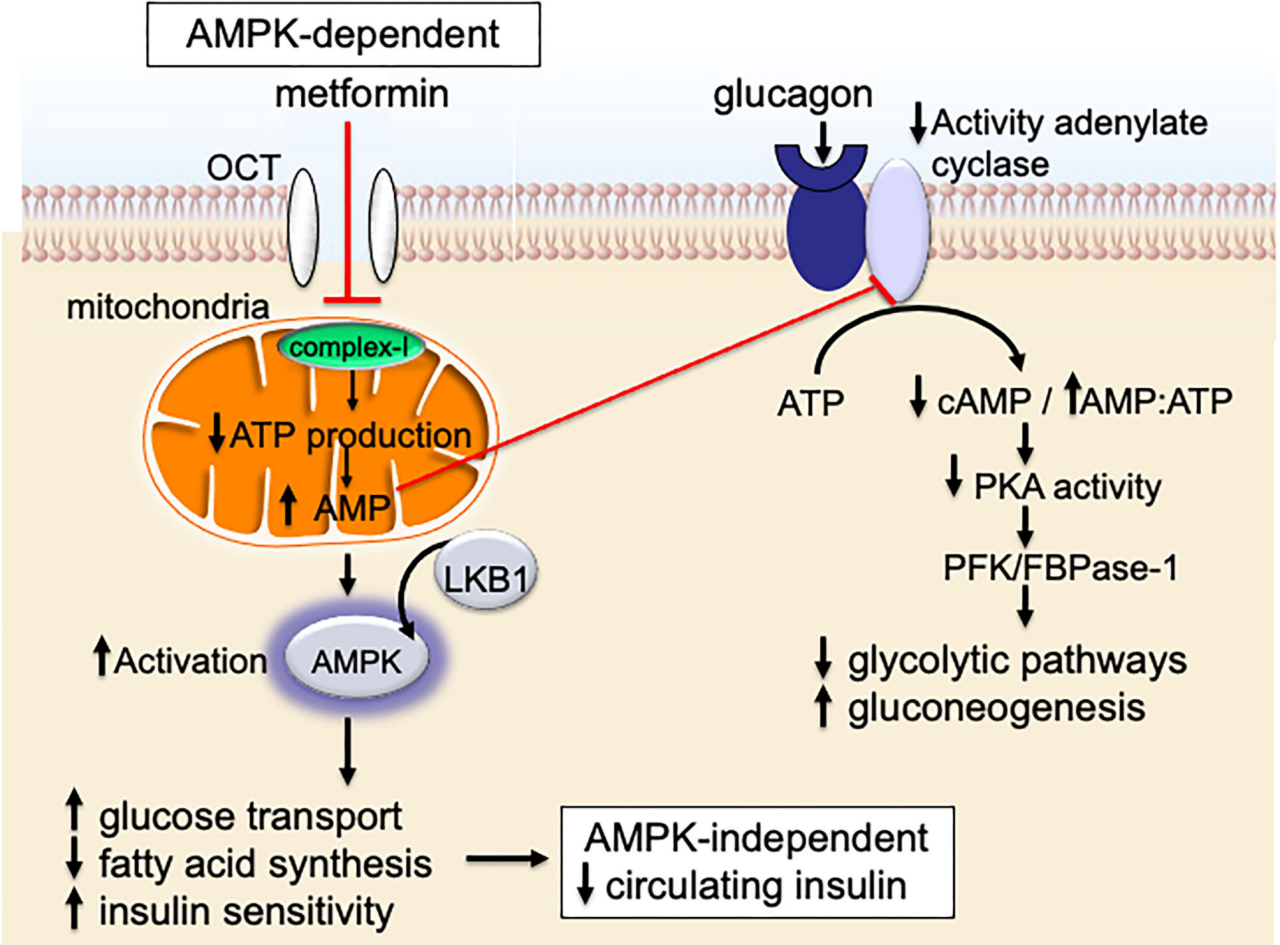
Metformin signaling in the liver.

Metformin also signals through an AMPK-dependent pathway; in this pathway, metformin first inhibits the mitochondrial electron transport protein complex-I (65, 66). Inhibition of complex-I, in turn, blocks production of mitochondrial adenosine-5′-triphosphate (ATP), increases the AMP/ATP ratio, results in a reduction of AMP, and lowers hepatic energy state [Figures 2, 3; (65–69)]. This hepatic energy state restriction leads to AMP binding to AMPK and, thereby, increasing AMPK's affinity for serine-threonine liver kinase B1 (LKB1) (70, 71). AMPK-LKB1-activaiton inhibits AKT/mTOR-network signaling leading to downstream inhibition of S6-Kinase (S6K) and 4E binding protein-1 (4EB-1). Metformin's inhibition of mTOR suppress additional downstream cancer-promoting pathways including (1) Nuclear Factor kappa-light-chain-enhancer of activated B cells NFkB/interleukin-6 (IL6), (2) MAPK/Ras, and (3) cMyc [Figure 2; (64, 72, 73)]. NFkB, IL6, MAPK, Ras, and cMyc together play a role in tissue inflammation, metabolism, and immune cell signaling.

Increasing attention has been paid to identifying molecular mechanisms that promote chemotherapy-resistance. Kevin Struhl's group first showed in 2009 that 0.1 mM metformin *in vitro* blocked transformation and killed cancer-like stem cells (74). The combination of metformin and doxorubicin in a mouse xenograft model (metformin 100 μg/ml) exhibited synergy. These results provided a potentially novel mechanism of action for metformin and an experimental rationale for using the combination of metformin and chemotherapy. The metformin doses in this study, however, were supratherapeutic and this very interesting mechanism of metformin-action remains an area of active investigation.

There is also evidence that metformin acts on the tumor microenvironment. Metformin increases intracellular oxygen; this increase is thought to reduce tumor hypoxia (75). Metformin's decrease in hypoxia has been shown to inhibit hypoxia-inducible factor 1 (HIF1) and vascular endothelial growth factor A (VEGFA) driven angiogenesis; there is also evidence for a direct anti-tumor effect on endothelial cells (76, 77). Metformin's increase in tumor oxygenation and or activation of AMPK is thought to shift cancer associated macrophages from a M2 to an M1 phenotype (78). Metformin has been shown to reduce programmed death-ligand 1 (PD-L1) expression on cancer cells, increase lymphocyte anti-tumor cytotoxicity, and downregulate myeloid derived tumor cell activity (79–82). Taken together, these findings highlight a potential role for metformin to be used in concert with immune-therapy.

### Current Consensus

While the study of metformin's molecular mechanisms of actions remain an area of active research, there is a growing consensus of the key signaling targets of metformin. The following consensus statement for metformin's key mechanisms of actions is updated from Pernicova and Korbonits (83):

- Metformin alters cellular energy metabolism and promotes metabolic reprogramming.
- Metformin acts to lower glucose and increase insulin-sensitivity: (1) primarily by inhibiting hepatic gluconeogenesis and glucagon-signaling and (2) to a lesser degree, in the skeletal muscle by increasing glucose uptake.
- Metformin lowers circulating glucose by inhibiting hepatic gluconeogenesis and opposing glucagon-action.
- Mitochondria complex-1 is a key target of metformin-signaling.
- Antihyperglycemic effect of metformin remains an area of active investigation, more work is needed.
- Metformin impacts lipid metabolism primarily via activation of 5′-AMP-AMPK.
- Anti-cancer effects of metformin are hypothesized to be: (1) indirect—decrease in circulating insulin and (2) direct—energetic stress. However, additional studies are needed.
- Metformin induces energetic stress in cancer cells.
- AMPK-mediation inhibition of mTOR is important for much of metformin's anticancer activity.
- Impact of metformin on cancer stem-like cells needs validation *in vivo* and in human clinical trials.
- Metformin may have direct and indirect anti-tumor effects on the tumor microenvironment.

## Rationale for Metformin's Ability to Prevent Biologically Aggressive Breast Cancers

**Figure 4:**
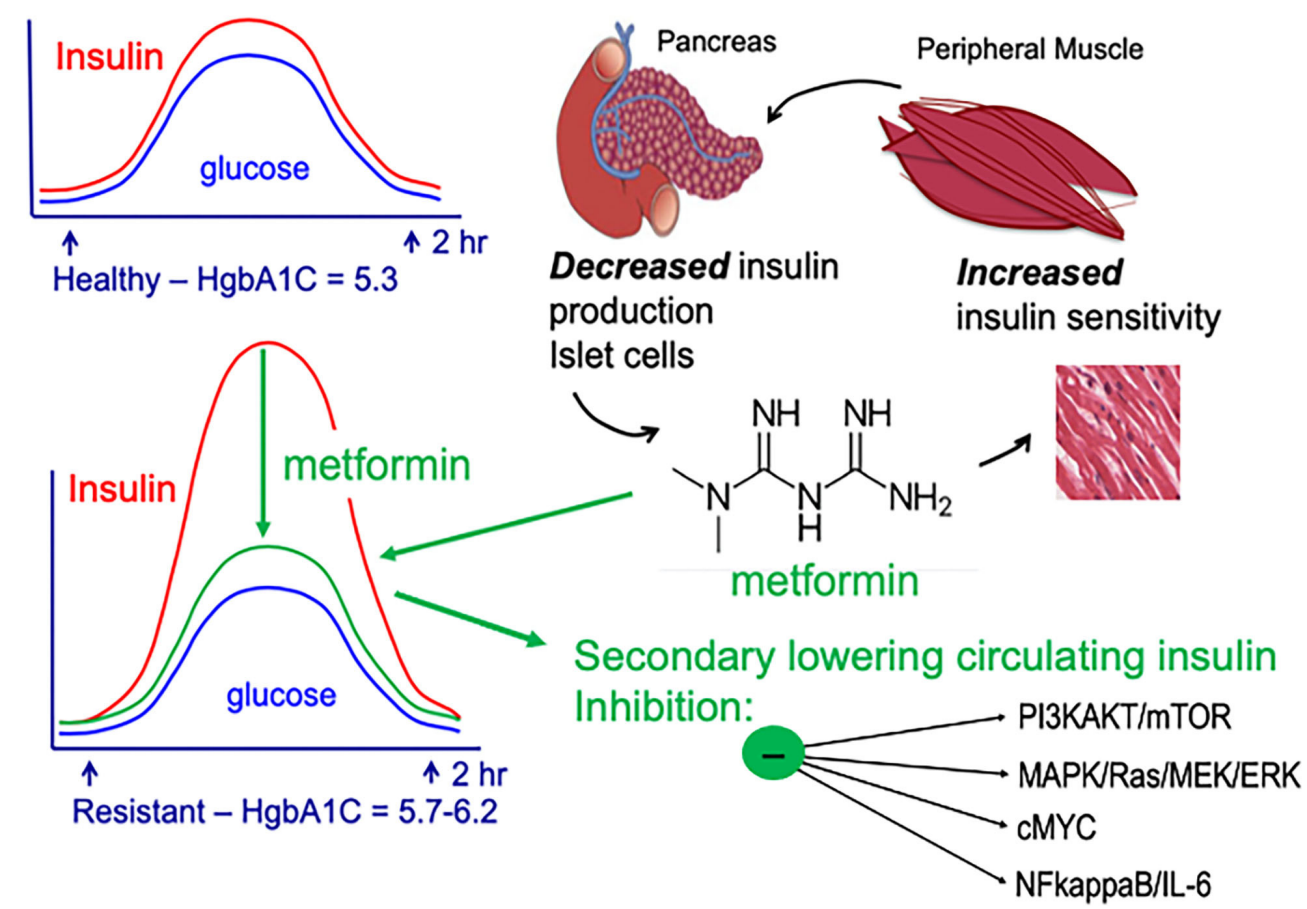
AMPK-dependent and AMPK-independent metformin signaling. Adapted from (42).

In breast cancer, particularly TNBC and basal-type breast cancer, activation of PI3K/AKT/mTOR-signaling pathway is associated with poor prognosis (84, 85). Activation of the PI3K/AKT/mTOR results in cell cycle progression, apoptosis-resistance, and invasion (86, 87). PI3K/AKT/mTOR is a regulator of glucose metabolism and aerobic glycolysis (Warburg effect) (88–90). The Warburg effect is directly linked to aggressive cancer biology due to its impact on glycolysis/glucose-uptake; increased glycolysis/glucose-uptake promotes increased growth, mitochondrial dysfunction, and apoptosis-resistance. Metformin targets the PI3K/AKT/mTOR pathway and promotes metabolic reprogramming. These actions support the use of metformin for prevention of biologically aggressive breast cancers (Figures 2–4).

Prevention options for premenopausal women who carry a deleterious germline BRCA mutation are limited. There is strong scientific rationale for testing metformin in chemoprevention of breast cancer in *BRCA* mutation carriers: (1) metformin activates AMPK and (2) signaling networks regulated by both AMPK and BRCA1, include PTEN, p53, and acetyl coenzyme A carboxylase alpha (ACCA) (83, 91, 92). AMPK regulates the phosphorylation/dephosphorylation cycles of ACCA (93, 94). Given that AMPK and BRCA1 both inactivate ACCA, it is hypothesized that metformin might compensate for BRCA1-loss. Further rational for metformin prevention in BRCA1 mutation carriers has been provided by Cuyas et al. (95). Introduction of BRCA1 mutation*185delAG* in MCF10A cells resulted in metabolic reprograming including (1) mitochondrial activation, (2) increased glucose- and glutamine-dependent activation of the tricarboxylic acid cycle (TCA), and (3) increased production of acetyl-CoA and malonyl-CoA (95). Metformin was shown *in vitro* to inhibit (1) mitochondrial biosynthetic capacity, (2) the TCA cycle, and (3) generation of lipogeneic precursors. The authors hypothesize that the ability of metformin to block (“starve”) mitochondrial-generated biosynthesis, might provide further rationale for using metformin for cancer prevention in women with germline *BRCA1*-mutation (95). As described below, to date, the epidemiologic and clinical trials using metformin have yielded conflicting results. The ability of metformin to prevent biologically aggressive breast cancers, particularly TNBC, requires the completion of the on-going prospective trials, such as MA-32.

### Clinical Studies

Dr. Pamela Goodwin has been a pioneer in the use of metformin for lowering insulin and breast cancer chemoprevention; she has developed some of the first trials testing metformin. In a trial of 32 women (4 dropout) with early stage breast cancer and fasting insulin of ≥45 pmol/L and glucose <7.0 mmol/L, administration of metformin 1500 mg per day for 6 months was associated with a 22.4% decrease in serum insulin [*p* = 0.024; (34)]. This study provided the rational for subsequent randomized clinical trials using metformin vs. placebo.

Window-of-opportunity trials provide important insight into metformin's mechanisms of action but have had conflicting results. In a Scottish trial, Hadad et al. tested the impact of metformin 500 mg ramp up and then 1,000 mg twice a day on Ki-67 and gene expression on 8 pilot women and a further 47 women with primary breast cancer; 7/32 women receiving metformin withdrew due to gastrointestinal upset (96). In women receiving metformin, Ki-67 fell significantly following metformin in both the pilot study (*p* = 0.041) and in the metformin arm (*p* = 0.027) but was unchanged in women who did not take metformin (96). Gene expression studies showed a decrease in mRNA expression in genes regulating AMPK; further analysis demonstrated that tumor necrosis factor receptor signaling, and mTOR- and AMPK-signaling were impacted by metformin (96).

The results by Hadad et al. contrast with a second window of opportunity trial. In a double-blind pre-surgical trial Bonanni et al. (2008-004912-10) randomized 200 non-diabetic women to metformin 850 mg/day vs. placebo for 4 weeks prior to surgery (97). Unlike findings by Hadad et al., Bonanni et al. observed no statistical difference in Ki-67 between arms (97). However, there was a differential impact on Ki-67 based on insulin-resistance (measured by homeostatic model assessment—HOMA). In women with HOMA >2.8 there was a 10.5% decrease in mean Ki-67 vs. an 11% increase in women with HOMA <2.8 (*p*-interaction = 0.045); women with Luminal B breast cancer had the greatest benefit [*p* = 0.005; (97)]. Further, biomarker analysis showed that this trial represented a significant accomplishment, given the difficulty of coordinating window-of-opportunity trials; importantly, this trial provided a key piece of evidence that non-diabetic metabolically unhealthy women may benefit from metformin chemoprevention (97). A third window-of-opportunity trial reported by Kalinsky et al. in women with early stage breast cancer and a BMI ≥30 reported that in women taking 1,500 mg metformin there were no significant differences in Ki-67 for either DCIS or invasive breast cancer (98). There has been significant discussion about the differences observed in these: trials; one potential difference is that women in the Scottish trial had larger breast cancers and therefore, had larger tumors for analysis [see Kalinsky and Hershman for a more in-depth analysis (99)]. Still, given the short duration of window-of-opportunity trials, longer duration trials with a cancer endpoint are required. See Table 2A for additional clinical and window-of-opportunity metformin trials in women with breast cancer.

**Table 2:** Review of metformin in breast cancer treatment or prevention.

**ClinicalTrials.gov (reference if available)**	**Study**	**Study design**	**Inclusion**	**Endpoint and results (if available)**
**(A) Adjuvant, window-of-opportunity, and secondary prevention trials**				
Breast phase II (34)	Insulin-lowering effects of metformin in women with early stage breast cancer	Metformin 500 mg tid ×6 months	IBC completed therapy with fasting insulin of ≥45 pmol/L and glucose <7.0 mmol/L	Serum insulin ***Results:*** Metformin was associated with a 22.4% decrease in serum insulin (*p* = 0.024)
NCT00897884 (100)	Clinical and biologic effects of metformin in early stage breast cancer	Window-of-opportunity. Single group. Metformin 500 mg tid ×3 weeks	Early stage disease. Women 18–70 years; T1-4; presurgical	Comparison pre- and post-operative biopsy; Ki67 ***Results:*** HOMA significantly reduced; Ki67 decreased 36.5–33% *p* = 0.016 TUNEL increased from 0.56 to 1.05 *p* = 0.004
NCT00909506	Efficacy and safety of adjuvant metformin for operable breast cancer patients	Window-of-opportunity. Metformin 500 mg ×1–2 weeks; then 500 mg bid weeks 3–24	Operable breast cancer BMI>23; no medications except tamoxifen	Weight loss
NCT00930579 (98)	Effects of metformin on AMP/mTOR pathway	Window-of-opportunity. Metformin 1,500 mg qd for >12 weeks before surgery	Operable breast cancer; BMI >30 overweigh and obese women with newly diagnosed breast cancer	***Results:*** No significant differences in Ki67 for DCIS or invasive breast cancer
NCT00933309 (101)	Impact of obesity and obesity treatments on breast cancer	Exemestane with metformin 1,000 mg per day and Rosiglitazone	Postmenopausal obese, ER+ metastatic breast cancer	Dose-limiting toxicity ***Results:*** Metformin was well-tolerated
NCT01042379	I-SPY 2 TRIAL: neoadjuvant and personalized adaptive novel agents to treat breast cancer	Window-of-opportunity. Randomized novel drugs in combination w/ standard chemotherapy	Presurgical breast cancer—neoadjuvant chemotherapy	Pathologic complete remission rate
NCT01101438 (MA-32) (102)	A phase III randomized trial of metformin vs. placebo in early stage breast cancer	Randomization to 1 of 2 treatment arms	Patients stratified by ER/PR status, BMI, HER2 status, and prior chemotherapy	Disease free survival Metabolic parameters: ***Results*** at 6 months: Weight −3.0%, glucose −3.8%, insulin −11.1%
NCT01310231 (103)	A trial of standard chemotherapy with metformin (vs. placebo) in women with metastatic breast cancer	Standard chemotherapy Metformin 850 bid vs. placebo	Metastatic breast cancer 1–4th line chemotherapy	***Results***: No significant impact on RR, PRS, or OS
NCT01650506	Study of Erlotinib and metformin in triple-negative breast cancer	Phase I to establish maximum tolerated dose	Open label single arm. Diagnosis of triple-negative breast cancer	Maximum tolerated dose
NCT01980823	Pre-surgical trial of the combination of metformin and atorvastatin in newly diagnosed operable breast cancer	Window-of-opportunity. Metformin 500 mg a.m. and 1,000 mg p.m. w/atorvastatin 80 mg or at least 2 weeks prior to surgery	Histologically confirmed DCIS or IBC who undergo CNB followed by surgery	Ki-67
NCT02145559 (104)	Pharmacodynamic study of sirolimus and metformin in patients w/advanced solid tumors	Pharmaco-dynamics study	Phase 1	Investigation of combination therapy in targeting mTOR pathway ***Results***: No dose limiting toxicities. No significant differences in fasting glucose, insulin, p70S6K
NCT02278965	Metformin and omega-3 fatty acids in women with a history of early stage breast cancer	Metformin 850 mg bid and Omega-3 1,120 mg bid ×12 months	Stage 1–3; no evidence of disease at entry	Safety and feasibility
NCT02874430	Metformin hydrochloride and doxycycline in treating patients with localized breast or uterine cancer	Metformin days 1–3; then 2x per day on day 4. Treatment repeats every 7 days	Breast or Uterine cancer; localized; no neoadjuvant chemotherapy	Increased caveolin in cancer associated fibroblasts
NCT03238495	Randomized trial of neo-adjuvant chemotherapy with or without metformin for HER2 positive operable breast cancer (HERMET)	Randomized taxotere, Carboplatin, Herceptin + Pertuzumab With or without metformin	cT1c-cT4a-d HER2+ breast cancer	Pathologic complete response
Instituto Europeo di Oncologica 2006-006236-22 (105)	Use of metformin to reduce serum level of testosterone and improve the metabolic picture for women treated with breast cancer	Metformin 1,000 vs. 1,500 mg/d ×3 months	Postmenopausal with history of IBC and 6 months post-surgery, on TAM for at least 6 months and plan to continue, or at least 6 months post-chemo	1,500 mg/d decreased testosterone by 23% (*p* < 0.01)
Instituto Europeo di Oncologica 2007-000306-70 (105)	Effect of metformin on biomarker activity in primary breast cancer.	Window-of-opportunity trial. Metformin 500 mg/d ×1 week; then metformin 1,000 mg/d ×1 week vs. placebo	Menopausal; Stage 1–2 IBC, >1 cm, no history of diabetes High risk of recurrence due to elevated testosterone	3.4% decrease in Ki-67 (*p* = 0.02)
Instituto Europeo di Oncologica 2008-004912-10 (97, 106, 107)	A randomized double-blind pre-surgical phase II study on activity of metformin on breast cancer cell proliferation	Window-of opportunity trial. Metformin 850 mg/d ×3 days; then metformin 850 mg bid day 4–28 vs. placebo; 4 weeks prior to surgery	Presurgical-Stage IIII IBC patient not suitable for neoadjuvant therapy	No overall change in Ki-67 10.5% decrease in Ki-67 if HOMA >2.8 (*p* for interaction = 0.045)
ClinicalTrials.gov **(reference if available)**	**Study title**	**Study design**	**Inclusion**	**Primary endpoint**
**(B) Primary prevention and presurgical trials**				
ACTRN 12610000219088	Phase I trial metformin followed by reduction mammoplasty	500 mg/d ×1 week; then 1,000 mg/d ×4 weeks; then reduction mammoplasty	Women age 40–60	AMPK signaling and aromatase expression in reduction mastectomy
NCT01302379 (108)	Reach for Health study: Obesity-related mechanisms and mortality in breast cancer survivors	Metformin Placebo Lifestyle interventions 2 ×2 design	Breast cancer survivor; no active disease Overweight or obese	Study powered for metformin vs. placebo and weight loss vs. control. Metformin associated with decrease in serum insulin, estradiol, testosterone
NCT01793948	Metformin hydrochloride vs. placebo in overweight and obese patients at elevated risk for breast cancer	850 mg qd ×30 days; then bid ×11 months vs. placebo	Postmenopausal and high risk for breast cancer with BMI ≥25	Changes in mammary epithelial phosphorylated proteins
NCT01905046	Metformin hydrochloride vs. placebo in preventing breast cancer in obese premenopausal women with atypical hyperplasia or *in situ* breast cancer	850 mg qd ×4 weeks; then 850 mg bid vs. placebo ×24 months	Premenopausal, BMI >25, prior AH, LCIS or DCIS, >1.66% Gail or known BRCA carrier, and cytological atypia	1^O^ Endpoint: Regression of atypia at 12 and 24 months 2^O^ Endpoint: Changes in phosphorylated proteins
NCT02028221	Phase II study of metformin for reduction of obesity-associated breast cancer risk	850 mg ×1 month; then 850 mg bid ×11 months vs. placebo	Premenopausal women age 30–45 with BMI of 25 or greater and metabolic syndrome	Change in breast density from baseline at 6 and 12 months
NCT02431676	Survivorship promotion in reducing IGF-1 trial	Metformin Coach directed behavioral weight loss Self-control weight loss	Breast cancer Prostate cancer Lung cancer	Serum IGF-1 IGF-1/IGFBP3 ratio
NCT04300790	Study to evaluate the effect of Metformin in prevention of hyperglycemia in HR+/HER2- PI3KCA-mutant advanced breast cancer patients [METALLICA]	Metformin Alpelisib Fulvestrant	Prevention hyperglycemia in cancer patients	Number of patients with grade 3–4 hyperglycemia

IBC, invasive breast cancer; DCIS, ductal carcinoma in situ; qd, one a day; bid, twice a day; tid, three times a day; Tam, Tamoxifen; BMI, body mass index; HOMA, Homeostasis Model Assessment; CNB, core needle biopsy; RR, recurrence rate; PFS, progression free survival; OS, overall survival.

*AH, atypical hyperplasia; LCIS, lobular carcinoma in situ; DCIS, ductal carcinoma in situ; qd, one a day; bid, twice a day; tid, three times a day; Tam, Tamoxifen; BMI, body mass index; RPPM, reverse phase proteomic microarray profiling*.

Currently many ongoing prospective clinical studies are testing the metformin for primary and secondary prevention of breast cancer (Tables 2A,B). Together, these clinical studies represent an important investment by the National Institute of Health, United States (NIH), European Cancer trials groups, and the National Cancer Institute, Canada (NCIC) (Table 2). The largest adjuvant (secondary prevention) trial is NCIC MA-32, comparing metformin 850 mg p.o. twice a day vs. placebo (NCT01101438) in women with breast cancer; the endpoint of this trial is breast cancer recurrence. After 2,382 women were enrolled, in 2012, the eligibility criteria were amended to mandate TNBC status for patients with T1cN0 disease and at least one adverse tumor characteristic for patients with T2N0 tumors. Interim analysis of the first 500 women taking metformin entered in MA-32, showed at 6 months there was a significant decrease in weight (−3.0%), serum glucose (−3.8%), and serum insulin (−11.1%) (102); further results from this trial are pending. ACTRN12610000219088 is currently testing the impact of metformin (1,000 mg) on LKB1 and AMPK signaling; NCT0430079 tests the impact of metformin in preventing grade 3–4 in (1) men and (2) post-menopausal women receiving treatment for ER/PR+, HER2-not amplified advanced breast cancer, with a PI3K-mutation [METALLICA trial]. Primary prevention studies include (1) NCT01793948: randomized testing the impact of metformin on postmenopausal women with high breast density, (2) NCT01905046: metformin vs. placebo in high-risk premenopausal women (including BRCA mutation carriers) with cytologic atypia, and (3) NCT01905046: randomized testing of whether metformin alters breast density, serum IGF-1/IGFBP-e ratios, IGF-2, and leptin/adiponectin ratios, body weight/body composition (109). See Table 2B for additional trials. Given the wealth of primary and secondary metformin chemoprevention trials, it is anticipated that over the next 5 years, these trials will provide important insights into whether metformin is a viable chemoprevention agent for breast cancer.

## Metformin and Heart-Healthy Prevention of Biologically Aggressive Breast Cancers

Metformin is cheap, safe during pregnancy, and has shown to prevent type-2 diabetes. There is a need for prevention drugs that target both ER+ and ER- breast cancer as well as providing prevention for cardiometabolic disease. Metformin clearly lowers insulin-signaling; signaling pathways activated by insulin are known to drive biologically aggressive breast cancer and predict poor survival in women with breast cancer. Despite the fact that metformin targets many key breast cancer pathways, there is much to be learned about whether metformin can prevent breast cancer and/or breast cancer recurrence. Window-of-opportunity trials provide important clues to metformin's impact on normal and malignant breast tissue, but results have not been entirely consistent. Currently, it is unclear which breast cancer subtypes may benefit the most from metformin. It is likely that MA-32 will provide answers to many of these questions. There is also much to be learned about metformin, insulin resistance, and BMI; specifically, whether metformin's impact is only in women who are metabolically unhealthy and/or have high BMI, or whether metformin can benefit all women. Biomarker studies that define key signaling pathways impacted by metformin will be critical to design and inform future clinical trials. Over the next 5 years on-going primary and secondary prevention trials will show (or not show) the ability of metformin to prevent breast cancer. Hopefully, these studies will not just provide a yes/no answer also provide the biomarkers to determine which women will maximally benefit from metformin. In the words of several of my patients “Please do not quote statistics at me; these statistics are about other women. If I take a prevention agent, I want to know if the prevention agent is working in my breasts.”

## Author Contributions

All authors listed have made a substantial, direct and intellectual contribution to the work, and approved it for publication.

## Conflict of Interest

The authors declare that the research was conducted in the absence of any commercial or financial relationships that could be construed as a potential conflict of interest.
